# Sumoylation is Required for the Cytoplasmic Accumulation of a Subset of mRNAs

**DOI:** 10.3390/genes5040982

**Published:** 2014-10-20

**Authors:** Hui Zhang, Kohila Mahadevan, Alexander F. Palazzo

**Affiliations:** Department of Biochemistry, University of Toronto, 1 King’s College Circle, MSB Room 5336, Toronto, ON M5S 1A8, Canada; E-Mails: huit.zhang@utoronto.ca (H.Z.); kohila.mahadevan@utoronto.ca (K.M.)

**Keywords:** RNAi, mRNA nuclear export, Ubc9, Sae1, sumoylation, GANP, mRNP

## Abstract

In order to discover novel proteins that promote the nuclear export of newly synthesized mRNAs in mammalian cells, we carried out a limited RNAi screen for proteins required for the proper cytoplasmic distribution of a model intronless mRNA. From this screen we obtained two hits, Ubc9 (SUMO-conjugating E2 enzyme) and GANP (germinal center-associated nuclear protein). Depletion of Ubc9 inhibited the proper cytoplasmic distribution of certain overexpressed intronless mRNAs, while depletion of GANP affected all tested mRNAs. Depletion of Sae1, which is also required for sumoylation, partially inhibited the cytoplasmic distribution of our model mRNA. Interestingly, the block in cytoplasmic accumulation in Ubc9-depleted cells could be overcome if an intron was incorporated into the mRNA. Surprisingly, Ubc9-depleted cells had normal nuclear export of newly synthesized intronless mRNAs, indicating that the observed accumulation of the model mRNA in the nuclei of transfected cells was likely due to some more general perturbation. Indeed, depletion of Ubc9, coupled with the overexpression of the intronless mRNAs, caused the redistribution of the nuclear speckle protein SC35 to cytoplasmic foci. Our results suggest that sumoylation may play a role in the proper assembly of mRNPs and/or the distribution of key RNA binding proteins, and may thus contribute to general protein expression patterns.

## 1. Introduction

Eukaryotic cells are divided into two compartments, the nucleoplasm, where mRNAs are synthesized, processed, and assembled into messenger ribonucleoprotein (mRNP) complexes, and the cytoplasm, where these mRNAs are translated into proteins. Nuclear export of mRNPs requires numerous different proteins and complexes which promote the export of fully processed mRNAs while inhibiting the export of intergenic RNAs and misprocessed mRNAs (for reviews see [1,2,3]). Although it has been proposed that certain processing steps, such as splicing, are required for nuclear export, it is clear that most intronless mRNAs (both natural and cDNA-derived) are also efficiently exported [2]. In contrast, most long non-coding RNAs are retained in the nucleus [4]. It is thus clear that we do not yet understand the rules governing whether a particular mRNA is a substrate for nuclear export, nor the proteins that differentiate between RNAs destined for the cytoplasm and those that should remain in the nucleus.

The major mRNA nuclear export complex in mammalian cells is the transcription export (TREX) complex. It is composed of the Tho complex, the RNA helicase UAP56 (or its paralog URH49), Aly, and other miscellaneous factors [2]. TREX recruits the nuclear transport receptor heterodimer TAP/p15 to the RNA, and this in turn ferries the messenger ribonucleoprotein (mRNP) complex across the nuclear pore. TREX can be recruited to the mRNA by a variety of processes [2]. The best studied mechanism operates upon the completion of splicing, when factors within the spliceosome help deposit TREX onto the 5'end of the mature mRNA [5,6]. Despite this, it is well known that certain TREX components are deposited onto and are required for the export of intronless mRNAs [7,8]. A second complex, TREX2, has also been implicated in the export of mRNAs in yeast [9]. TREX-2 associates with the nuclear pore [10,11,12,13,14], and at least one component of this complex, germinal center-associated nuclear protein (GANP), has been implicated in the export of mRNAs in mammalian cells [12,13,14].

Although screens for nuclear mRNA export components have been performed in many genetically tractable systems, such as in yeast [15,16] and insect cells [17,18,19], few screens have been carried out in mammalian systems. Indeed, certain factors that appear to be critical for export in Drosophila S2 cells are dispensable for mRNA nuclear export in human cells, RanBP2/Nup358 being one example [20,21]. Other key nuclear export factors have undergone gene duplication to produce several proteins that act redundantly to promote export in mammals, a prime example being the RNA helicases UAP56 and URH49 [22,23]. Additional factors such as Aly and the Tho complex only produce effects when they are depleted in combination [24].

Here we conducted a screen for genes that are required for the efficient cytoplasmic steady-state distribution of a model intronless mRNA, *MHC-ftz-*Δ*i*.

## 2. Experimental Section

### 2.1. Plasmids

Plasmids used were *ftz* reporter construct, human *insulin* cDNA [23] or human β*-globin-i* [8] in pCDNA3.1, human *INSL3*, *CALR*, or *ALPP* cDNAs in pSPORT6 and *H1B-GFP* in pEGFP [25]. Except for *ftz*, *H1B-GFP* and β*-globin-i*, the constructs contained the natural open reading frames and complete untranslated regions. ShRNA plasmids (details below) were purchased from Sigma.

### 2.2. Lentiviral Mediated shRNA Knockdown

Plasmids encoding shRNA against Ubc9 (TRCN0000320448, hairpin sequence CCGGAGCAGAGG CCTACACGATTTACTCGAGTAAATCGTGTAGGCCTCTGCTTTTTTG and TRCN0000368347, sequence CCGGAGAAGTTTGCGCCCTCATAAGCTCGAGCTTATGAGGGCGCAAACTTCTTTTTTG), Sae1 (TRCN0000007475, sequence CCGGGCTATGTTGGTCCTTTGTTTACTCGAGTAAACAAAGGACCAACATAGCTTTTTG and TRCN0000007477, sequence CCGGGAACAGGTAACTCCAGAAGATCTCGAGATCTTCTGGAGTTACCTGTTCTTTTTG), GANP (TRCN0000146606, sequence CCGGCAGGAGCATTTACGGATTTAACTCGAGTTAAATCCGTAAATGCTCCTGTTTTTTG and TRCN0000147338, sequence CCGGGAAACTCATCTGAGGTGAATCTCGAGATTTCACCTCAGATGAGTTTCTTTTTTG), RNPS1 (TRCN0000000049, sequence CCGGCGTAGAGTTTGAGAATCCAGACTCGAGTCTGGATTCTCAAACTCTACGTTTTT and TRCN0000320710, sequence CCGGCAGCTCCAACTCCTCCCGATACTCGAGTATCGGGAGGAGTTGGAGCTGTTTTT), U2AF65 (TRCN0000314892, sequence CCGGGAAGACCAGTAGGAAAGCAAACTCGAGTTTGCTTTCCTACTGGTCTTCTTTTT and TRCN0000314894, sequence CCGGCAGCTCCAACTCCTCCCGATACTCGAGTATCGGGAGGAGTTGGAGCTGTTTTTG) or empty vector (pLKO.1) were transfected into the HEK293T cells together with the accessory plasmids, VSVG and Δ8.9, to generate lentivirus carrying specific shRNA plasmids. Lentivirus was harvested from the supernatant medium 48 h post-transfection by filtering through a 0.44 µm filter. For infection, lentivirus-containing medium was applied to U2OS cells with 8 µg/mL hexadimethrine bromide. Puromycin was applied to the cells 24 h post-infection at 2 µg/mL to select for infected cells, and puromycin-containing medium was changed every other day. Cell lysates were collected five days post-infection to assess the level of knockdown, and the cells were used for downstream applications as described.

### 2.3. Cell Culture and Transfection

Cell culture and transfection were carried out as described previously [21,26]. Briefly, U2OS cells were maintained in Dulbeco’s Modified Eagle Medium (DMEM) supplemented with 10% fetal bovine serum. Cells were plated overnight on 35-mm-diameter dishes or acid washed 1.5 coverslips, transfected with 1 μg of the plasmid of interest, using GenJet *in vitro* transfection reagent for U2OS cells (SignaGen Laboratories, Gaithersburg, MD, USA) following the manufacturer’s protocol, and incubated for 18–24 h.

### 2.4. Fluorescent in Situ Hybridization (FISH) and Indirect Immunofluorescence

The procedure for fluorescent *in situ* hybridization FISH and immunofluorescence staining were carried out as previously described [8,21,26]. The nuclear speckle marker SC35 was immunostained with a primary antibody against SC35 (SC35 mouse monoclonal; 1:1000; Sigma product No. S4045) and a secondary antibody (Alexa647-conjugated donkey anti-mouse polyclonal; 1:1000; Life Technologies, Carlsbad, CA, USA). *Ftz*, *ALPP*, *CALR*, *INS*, *INSL3*, β*-globin* and Oligo dT DNA specific probes were performed as described previously [8,21,23,27]. Samples were mounted on Fluoromount with 4',6-diamidino-2-phenylindole (DAPI) (Southern Biotechnologies, Birmingham, AL, USA). Microscopy, imaging and quantification were performed as described previously [26]. All images were captured by epifluorescence on a TI-E inverted Nikon Microscope using a 60X phase 2, oil objective and a Coolsnap HQ2 14 Bit CCD camera (Photometrics, Tucson, AZ, USA) controlled using NIS elements Basic Research Microscope Imaging software [28]. Image exposures varied from 20 ms to 1 s. When analyzing protein and mRNA levels and distribution, all samples in a given experiment were imaged in a single sitting, and the exposure times were fixed between samples. From the raw unprocessed images, total integrated intensity, cytoplasmic/total, and nuclear/total ratio were calculated as described previously [26]. For figure preparation, the contrast and brightness of micrographs were adjusted in Photoshop [29].

### 2.5. Microinjection

U2OS cells were microinjected as previously described [26] with *MHC-ftz-∆i* plasmid (pcDNA3) DNA (200 ng/μL), 70kDa FITC-dextran (Life Technologies, Carlsbad, CA, USA) in injection buffer (10 mM HEPES, pH 7.4, 100 mM KCl). Cells were then incubated at 37 °C for various durations, then washed three times with PBS, and fixed with 4% paraformaldehyde in PBS for 15 min.

### 2.6. Northern Blots

After 18–24 h of transfection, total RNA was extracted from cells using PureLink RNA Mini Kit (Life Technologies, Carlsbad, CA, USA) following the manufacturer’s protocol. RNA was separated on a denaturing agarose gel, transferred, and probed for *ftz* and *GFP* as previously described [21].

### 2.7. Western Blots

Cell lysates were collected in 1× Laemmli sample buffer and resolved by SDS-PAGE on 10% gel. Gels were transferred to nitrocellulose and immunoprobed with antibodies against Ubc9 (rabbit polyclonal; 1:1000, Cell Signaling), αTubulin (mouse monoclonal DM1A; 1:10,000; Sigma, Aldridge, St. Louis, MO, USA), GANP (Rabbit polyclonal, 1:50; Assaybiotech, Sunnyvale, CA, USA), U2AF65 (mouse monoclonal MC3; 1:1000; Sigma, Aldridge, St. Louis, MO, USA), RNPS1 (1:2000; [30]) and/or Sae1 (rabbit polyclonal; 1:1000, GeneTex, Irvine, CA, USA) and with appropriate secondary antibodies conjugated to HRP (Life Technologies, Carlsbad, CA, USA). Presence of secondary antibody was detected by ECL reagent (Thermo Fisher Scientific, Rockford, IL, USA).

## 3. Results

### 3.1. A Screen to Identify Genes Required for mRNA Nuclear Export

**Figure A1:**
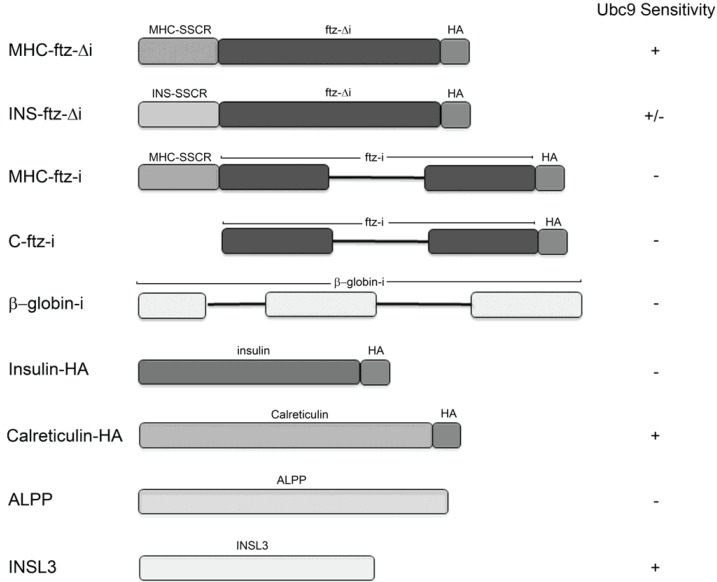
List of model mRNA transcripts used in this study. Note that the ability to accumulate in the cytoplasm in a Ubc9 dependent manner (“Ubc9 Sensitivity”) is indicated on the right.

Although many reports have surfaced in the literature claiming to identify novel components required for mRNA nuclear export in mammalian cells, few of these reports have been verified independently. To screen these factors, we employed the model mRNA *MHC-ftz-*Δ*i* (Figure A1). This engineered construct contains the *Drosophila melanogaster* *ftz* mini-gene, which has been used extensively as a model for splicing and mRNA nuclear export in vertebrate cells [23,31,32,33,34]. Since many groups have used the *ftz* mRNA in export studies, it is one of the best-characterized mRNAs. The particular version of *ftz* used in our screen lacks an intron and contains a signal sequence coding region (SSCR) derived from the mouse major histocompatibility complex (MHC) class II gene (Figure A1). SSCRs are present at the 5'end of the open reading frame (ORF) and encode hydrophobic alpha-helices that target the newly synthesized protein to the endoplasmic reticulum [35]. This specific SSCR is depleted of adenines, has CUG-repeats and has a high GC-content, three features that help it to promote the alternative nuclear mRNA export (ALREX) of microinjected mRNA [23,34].

Although we and others had previously reported that *ftz* requires splicing or an SSCR to be exported [23,32], we have recently discovered that this gene contains a negative export element [36]. In the absence of this element, the mRNA is efficiently exported to the cytoplasm without the need of an ALREX-SSCR or splicing, provided that it is transcribed *in vivo* (*i.e.*, not introduced as an exogenous mRNA molecule into the cell by microinjection). It remains unclear whether *ftz* contains additional elements that promote nuclear export, or whether other intronless mRNAs known not to be exported (such as human β-*globin*, *XSmad* and *AdML* [2,37,38]) contain elements that inhibit export. The presence of export-inhibiting elements in certain transcripts may explain why many artificial intronless mRNAs are efficiently exported from the nuclei of mammalian cells [2], presumably because they lack such inhibitory elements.

**Figure 1:**
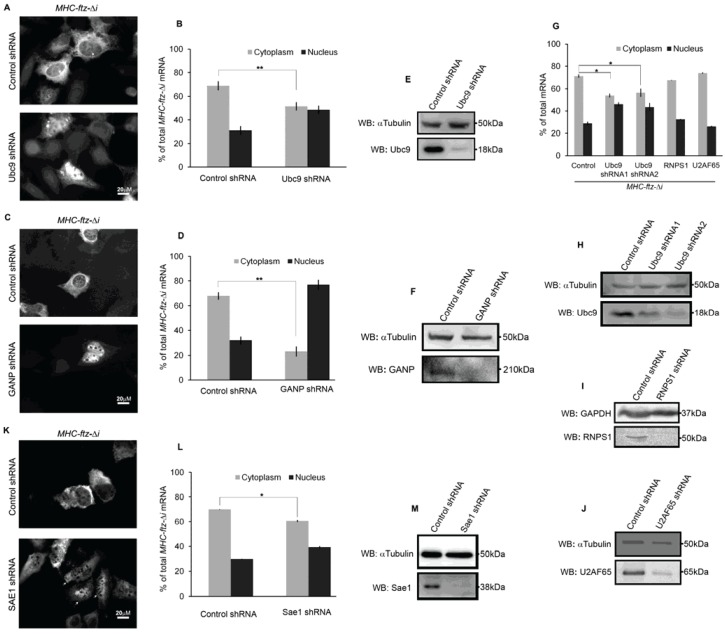
Ubc9, germinal center-associated nuclear protein (GANP) and Sae1 depletion alter the distribution of reporter mRNA *MHC-ftz-*Δ*i.* U2OS cells were treated with lentiviruses which delivered plasmids containing control shRNA or pairs of shRNAs against Ubc9 (**A**, **B**, **E**), GANP (**C**, **D**, **F**), RNPS1 (**G**, **I**), U2AF65 (**G**, **J**) or Sae1 (**K**–**M**). Single shRNAs against Ubc9 were used in (**G**, **H**). Three (GANP) or four (Ubc9, RNPS1, U2AF65 and Sae1) days post-infection, cells were transfected with *MHC-ftz-*Δ*i* reporter plasmid. The cells were then allowed to express the mRNA for 18–24 h. Transfected cells were then fixed and stained with a specific fluorescence in situ hybridization (FISH) probe against *ftz* sequence. Representative images (**A**, **C**, **K**) and the quantification summary of the cytoplasmic and nuclear integrated fluorescence intensity (**B**, **D**, **G**, **L**) of *MHC-ftz-*Δ*i* mRNA are shown in control and depleted cells. Each bar indicates the average and standard error of three independent experiments in which each experiment consists of an average of at least 40–70 cells. * *p* < 0.05, ** *p* < 0.005. (**E**, **F**, **H**–**J**, **M**) Lysates of cells were probed with antibodies against Ubc9, GANP and Sae1. Immunoblotting against αTubulin or glyceraldehyde 3-phosphate dehydrogenase (GAPDH) was used as a loading control.

We compiled a list of genes that had been reported to be required for mRNA nuclear export in a variety of systems, totaling 60 genes (Table S1). For each candidate we generated a pool of lentivirus that contained plasmids from which two separate shRNAs were produced to target and downregulate the gene of interest. Each pool was then used to infect human osteosarcoma (U2OS) cells. Three to four days post-infection, the cells were transfected with a plasmid that contained the *MHC-ftz-*Δ*i* reporter. The mRNA was allowed to express for 18–24 h, and its steady state levels were assessed by fluorescent *in situ* hybridization (FISH). To ensure reproducibility, this procedure was repeated at least three times. Normally the steady state distribution of *MHC-ftz-*Δ*i* is about 70% cytoplasmic, and we considered any positive hit as lowering this level to below 55%. The only two targets whose depletion caused a consistent change in mRNA distribution across all experiments were Ubc9 and GANP (Figure 1A–D). To ensure that the intended targets were depleted, we assessed protein levels by western blot. Both proteins were effectively depleted five days post infection (Figure 1E,F).

Ubc9 is the only known SUMO E2 conjugating enzyme in human cells [39]. SUMO is a small ubiquitin-like moiety that is covalently attached to the lysines of a substrate protein. Sumoylation may act to alter either the distribution or activity of a substrate protein. Although sumoylation has yet to be linked to mRNA export in mammalian cells, it has been proposed to play such a role in plant cells [40]. GANP is the human ortholog of the yeast nuclear export factor Sac3, a member of the TREX2 complex. GANP has been shown to be required for proper mRNA export in mammalian cells [12,13,14]. In our experiments, we consistently saw that depletion of GANP had a larger effect on *MHC-ftz-*Δ*i* mRNA distribution than depletion of Ubc9 (compare Figure 1B–D).

To confirm our Ubc9 result, we repeated the experiment with single shRNAs. Depletion of Ubc9 with either of the two shRNAs alone also inhibited the cytoplasmic accumulation of *MHC-ftz-*Δ*i* mRNA (Figure 1G,H). In contrast, depletion of U2AF65, which had previously been found to be important for the export of intronless mRNAs [7], or depletion of RNPS1, which has been linked to mRNA export in *Drosophila* [17] and mammalian cells [41], had no effect (Figure 1G,I,J).

Since Ubc9 is the only known SUMO E2 ligase in the human genome, we next tested whether depletion of another essential component of the sumoylation pathway had similar effects on the distribution of *MHC-ftz-*Δ*i* mRNA. Free SUMO is first activated by a heterodimer E1 ligase consisting of Sae1 and Sae2. The activated SUMO is then transferred to Ubc9, which then acts in concert with various E3 ligases to attach SUMO onto the lysines of a target substrate. With this in mind, we depleted Sae1 and assessed the distribution of *MHC-ftz-*Δ*i* mRNA. Although the distribution of the mRNA was not as effected as in Ubc9-depleted cells, a subset of Sae1-depleted cells had an increase in nuclear *MHC-ftz-*Δ*i* mRNA, (Figure 1K, see arrows). Overall this led to a slight decrease in cytoplasmic/nuclear ratio for this mRNA (Figure 1L). Sae1 was efficiently depleted as detected by western blot (Figure 1M).

From these experiments we conclude that GANP, Ubc9, and Sae1 are required for the proper steady-state distribution of exogenously expressed *MHC-ftz-*Δ*i* mRNA in the cytoplasm. Although it is possible that the shRNAs may have had off-target effects, other groups’ observations that GANP affects bulk mRNA export and that both Ubc9 and Sae1 act in the same pathway (*i.e.*, sumoylation) make it highly likely that the effects are due to the depletion of the intended targets. The idea that this assay yields few off-target hits which impact export is supported by data showing that other shRNAs tested (~60 pairs of shRNAs) had no effect on the distribution of *MHC-ftz-*Δ*i* mRNA in the cytoplasm (Table S1). Depletion of Sae1 may have had less of an effect on mRNA distribution than depletion of Ubc9 since these acted enzymatically. Low levels of each protein may affect total sumoylation capability differently, ultimately leading to various degrees of inhibition on mRNA nuclear export.

### 3.2. GANP, But Not Ubc9, Is Required for the Normal Cytoplasmic Distribution of Bulk mRNA

**Figure 2:**
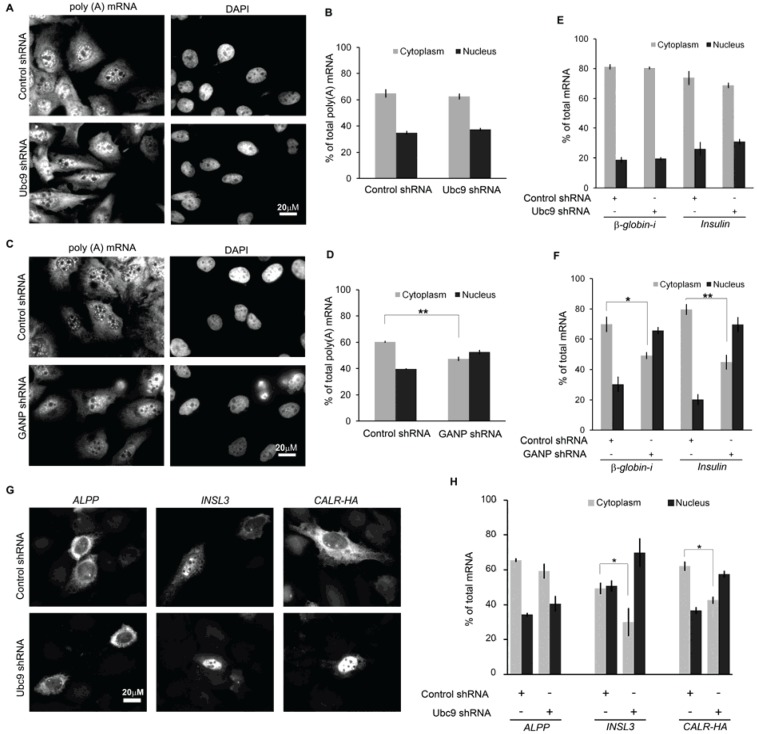
The effect of Ubc9 and GANP depletion on the distribution of poly(A) mRNA and other model transcripts. U2OS cells were infected with lentiviruses, which delivered plasmids containing control shRNA or pairs of shRNAs against Ubc9 or GANP, as in Figure 1A–D. Four (GANP) or five (Ubc9) days post-infection, cells were stained with oligo-dT probes and 4',6-diamidino-2-phenylindole (DAPI) as described previously [27]. Representative examples (**A**, **C**) and quantification summary (**B**, **D**) of the poly(A) mRNA distribution by FISH are shown. Note that each row in A and C represents a single field of view imaged for poly(A) and DNA. Each bar is an average and standard error of three independent experiments, each consisting of 60–90 cells. (**E**–**H**) Depleted cells were transfected as in Figure 1, but this time with plasmids containing either β*-globin-i*, an intronless version of *insulin*, *placental alkaline phosphatase* (*ALPP*), *insulin-like 3* (*INSL3*) or HA-tagged *calreticulin* (*CALR-HA*). After allowing expression for 18–24 h, cells were fixed and stained with specific FISH probes against each mRNA. Representative examples (**G**) and quantification summaries (**E**, **F** and **H**) of the cytoplasmic and nuclear integrated fluorescence intensity for each mRNA are shown, with each bar representing an average of three independent experiments in which 40–60 cells were used in each experiment. * *p* < 0.05, ** *p* < 0.005.

To determine how the depletion of these proteins affects overall mRNA distribution, depleted cells were stained for total poly(A) mRNA. Depletion of Ubc9 did not alter the distribution of bulk mRNA (Figure 2A,B). As reported previously [12,14], depletion of GANP led to an accumulation of nuclear poly(A) signals (Figure 2C,D). Next we examined two other model mRNAs, β*-globin-i*, which is exported in a splicing dependent manner [2,37], and an intronless version of *insulin* which requires its SSCR for export [23]. The steady-state distribution of both of these mRNAs was affected by the depletion of GANP and not Ubc9 (Figure 2E,F).

To determine whether Ubc9 could affect the distribution of other intronless mRNAs with ALREX-SSCRs, we investigated additional genes. Ubc9-depletion reduced the cytoplasmic distribution of *calreticulin* (*CALR-HA*) and *insulin-like 3* (*INSL3*), but not *placental alkaline phosphatase* (*ALPP*) mRNA (Figure 2G,H, for information on these constructs, see Table S1). Importantly, all three of these mRNAs have features associated with ALREX (SSCRs with low adenine-content, CUG-repeats, and high GC-content) [34] and in the case of *CALR-HA*, the adenine-depletion in the SSCR has been shown to potentiate translation of the mRNA in *cis* [21]. These results suggest that Ubc9-sensitivity is not related to the presence of an SSCR in a given mRNA.

From these experiments we conclude that the distribution of only a select number of mRNAs was affected by Ubc9 depletion. In contrast, GANP appears to be required for the proper export of many more mRNAs.

### 3.3. Splicing Can Re-Establish the Normal Cytoplasmic Distribution of ftz mRNA in Ubc9-Depleted Cells

**Figure 3:**
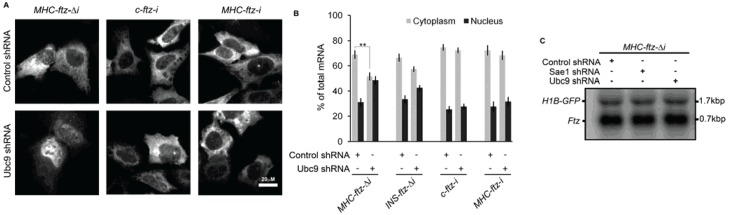
Ubc9-depletion has no effect on the export of spliced *ftz* mRNA and does not affect the level of unspliced *MHC-ftz-*Δ*i* mRNA. U2OS cells were treated with lentivirus containing shRNAs targeting Ubc9 or control shRNA as in Figure 1. Four days post-infection, cells were transfected with plasmids containing the indicated versions of the *ftz* gene. After 18–24 h cells were fixed and stained with specific FISH probes against the exogenously expressed mRNA. (**A**) Examples of cells expressing the indicated *ftz* mRNAs; (**B**) Quantification of the nuclear/cytoplasmic distribution of the various mRNAs. Each bar is the average and standard error of three independent experiments, each consisting of 50–80 cells. * *p* < 0.05, ** *p* < 0.005; (**C**) RNA was isolated from depleted cells, separated on a denaturing agarose gel and detected using specific 32P-labeled DNA probes against *ftz* and *GFP*.

In an attempt to determine what features govern Ubc9-sensitivity, we assessed the distribution of various versions of the *ftz* mRNA in knockdown cells. Interestingly, the inclusion of an intron (*i*) into the construct, regardless of whether (*MHC-ftz-i*) or not (*c-ftz-i*) there was an ALREX-promoting SSCR (for information on these constructs see Table S1), completely abolished Ubc9 sensitivity (Figure 3A,B). This intron is known to be efficiently spliced in mammalian cells [23] and enhances the association of the nuclear export-promoting complex TREX with the mature mRNA [6]. *INS-ftz-*Δ*i* mRNA, which contains the *insulin* SSCR, appeared to be less sensitive to Ubc9 depletion than *MHC-ftz-*Δ*i*, although this effect was not statistically significant (Figure 3B).

It is thus clear from these experiments that splicing abolishes the sensitivity of the *ftz* mRNA to Ubc9-depletion. However it still remains unclear why only a subset of intronless mRNAs shows this sensitivity.

### 3.4. MHC-ftz-Δi Expression Levels Are Normal in Ubc9- and Sae1-Depleted Cells

It has been reported that sumoylation modulates the activity of the 3'end processing and polyadenylation complexes [42]. It is also possible that sumoylation may modulate transcription and/or mRNA turnover. However, depletion of either Ubc9 or Sae1 did not have any major effects on either the apparent size or the amount of *MHC-ftz-*Δ*i* mRNA, as judged by northern blot (Figure 3C). We also examined the levels of mRNA from a co-transfected plasmid-containing *histone 1B-GFP* (*H1B-GFP*) to control for transfection efficiencies, and again saw no major changes. From this we conclude that sumoylation does not affect the expression level of *MHC-ftz-*Δ*i*. Although mRNA processing does not appear to be grossly affected in Ubc9-, and Sae1-, depleted cells, it remains possible that the polyadenylation of *MHC-ftz-*Δ*i* mRNA may be altered to a level undetectable by our northern blot analysis.

### 3.5. Depletion of GANP, but not Ubc9, Inhibits the Nuclear Export of Newly Synthesized MHC-ftz-Δi mRNA

**Figure 4:**
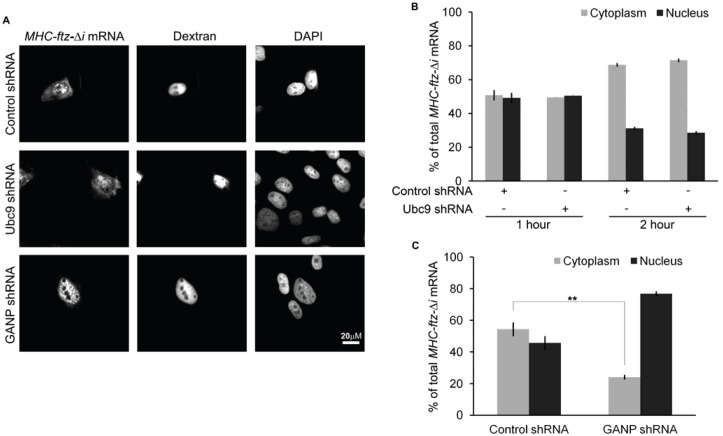
GANP, but not Ubc9, is required for the export of newly synthesized mRNA. U2OS cells were treated with lentivirus containing shRNAs targeting Ubc9, GANP or control shRNA as in Figure 1. Four (GANP) or five (Ubc9) days post-infection, the cells were microinjected into the nucleus with *MHC-ftz-*Δ*i* reporter plasmid DNA and allowed to express the mRNA for 20 min. To mark microinjected cells, Alexa488-conjugated 70 kD dextran was included in the microinjected fluid. The cells were then treated with α-amanitin to halt further transcription, and the cells were allowed to export the newly synthesized mRNA for either 1 (**A**–**C**) or 2 (**B**) h. The cells were then fixed and stained for mRNA using specific FISH probe against *ftz*. Each row represents a single field of view imaged for *ftz* mRNA, Alexa488-dextran and DAPI. Quantification of the nuclear/cytoplasmic distribution of the various mRNAs is shown. Each bar is the average and standard error of three independent experiments, each consisting of at least 30 cells. ** *p* < 0.005.

A disruption in the steady-state distribution of mRNA to the cytoplasm can be the end result of many different perturbations. One way in which this can occur is by the disruption of normal mRNA nuclear export. To test this directly, we employed a microinjection assay where the export of newly synthesized mRNA was assessed [26]. This involved microinjecting plasmids directly into nuclei, allowing transcription to proceed for 20 min, and then treating the cells with α-amanitin to halt further mRNA production. Nuclear export of the newly made mRNA was then allowed to proceed for various times, after which the samples were fixed. The distribution of the mRNA at each time point was monitored by FISH. To our surprise, Ubc9-depletion had no measurable effect on the export of *MHC-ftz-Δi* mRNA, and this was true at both the 1 and 2 h time points (Figure 4A,B). In contrast, depletion of GANP inhibited the nuclear export of newly synthesized *MHC-ftz-*Δ*i* (Figure 4C,D).

From this result we conclude that sumoylation does not play a direct role in regulating mRNA nuclear export. In contrast, GANP is likely to directly mediate the export of newly synthesized *MHC-ftz-*Δ*i* mRNA.

### 3.6. Overexpression of MHC-ftz-Δi in Ubc9-Depleted Cells Disrupts Nuclear Speckles

**Figure 5:**
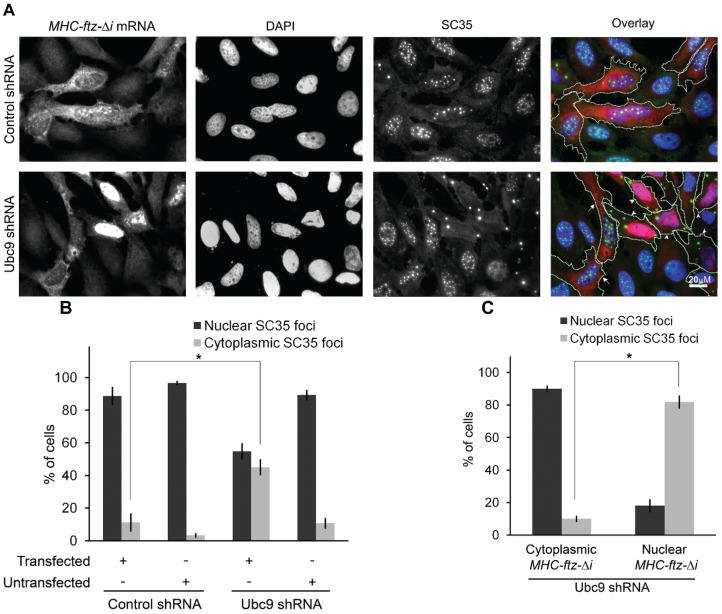
Expression of *MHC-ftz-*Δ*i* in Ubc9-depeled cells causes SC35 mislocalization. U2OS cells were treated with lentivirus containing shRNAs targeting Ubc9 or control shRNA as in Figure 1. Four days post-infection, the cells were transfected with *MHC-ftz-*Δ*i* reporter plasmid DNA. The cells were allowed to express the mRNA for 18–24 h; then they were fixed and stained for *ftz* mRNA by FISH and SC35 by immunofluorescence. (**A**) Representative images, including an overlay of *MHC-ftz-*Δ*i* (red), DNA (stained by DAPI, blue), and SC35 (green). The transfected cell boundaries are outlined with white dotted lines. Arrows indicate cells with cytoplasmic *MHC-ftz-*Δ*i* mRNA, while arrowheads mark cells with nuclear *MHC-ftz-*Δ*i* mRNA. Scale bar = 20µm; (**B**) The quantification summary shows the percentage of transfected and untransfected cells with SC35 speckles in the nucleus or the cytoplasm in the control and Ubc9 knockdown cells. Each bar consists of an average of three independent experiments, each consisting of at least 125 cells. * *p* < 0.05; (**C**) Transfected cells from (**B**) were split into two groups, those that had high and low ratios of cytoplasmic/nuclear *MHC-ftz-*Δ*i* mRNA. Each group was quantified for the distribution of SC35 foci.

Previously we had found that inhibition of nuclear export, through either depletion of UAP56/URH49 or inhibition of TAP, caused an accumulation of mRNA in nuclear speckles [8], large nuclear foci that contain splicing factors and TREX components [43]. We also noticed that many mRNAs which were efficiently exported were also trafficked through these structures [8]. This observation was consistent with the finding that certain TREX components were loaded onto mRNA either within or in the vicinity of these structures [44,45]. Intriguingly, in Ubc9-depleted cells, *MHC-ftz-*Δ*i* mRNA that was retained in the nucleus did not accumulate in foci (for example, see Figure 1A and Figure 3A), suggesting that they were not targeted to nuclear speckles. We therefore decided to visualize both mRNA and SC35, a well-known marker of nuclear speckles [46,47]. Surprisingly, many of the Ubc9-depleted cells that expressed *MHC-ftz-*Δ*i* had lost all of their nuclear SC35 foci. Instead, SC35-positive aggregates were present in the cytoplasm (Figure 5A, for quantification, see Figure 5B). Depleted cells, which did not express *MHC-ftz-*Δ*i*, did not display this phenotype. Upon closer inspection we noticed that it was only the Ubc9-depleted cells with a nuclear accumulation of *MHC-ftz-*Δ*i* mRNA that had cytoplasmic SC35 foci (Figure 5A, cells with nuclear *MHC-ftz-*Δ*i* are denoted by arrowheads; for quantification see Figure 5C). In contrast, Ubc9-depleted cells that had robust nuclear export of *MHC-ftz-*Δ*i* mRNA also had normal distributions of SC35 (Figure 5A, cells with exported *MHC-ftz-*Δ*i* are denoted by arrows; for quantification see Figure 5C). Various cytoplasmic RNA granules, such as P-bodies and stress granules, are known to be associated with mRNA degradation [48]; however, SC35 foci did not colocalize with the P-body component GW182, or the stress granule marker TIA-1 [49], suggesting that these foci were not sites of mRNA decay.

From these observations we conclude that in cells depleted of sumoylation factors, the overproduction of certain mRNAs causes the reorganization of certain nuclear speckle markers to the cytoplasm. Likely this nuclear-cytoplasmic reorganization of SC35 and potentially other nuclear speckle components is causing mRNA export to grind to a halt.

## 4. Discussion

Here we demonstrate that cells depleted of SUMO-conjugating enzymes display a defect in the cytoplasmic accumulation of certain overexpressed intronless genes. This is the first report, to our knowledge, linking sumoylation to mRNA nuclear export in metazoan cells. Sumoylation has, however, been linked to mRNA export in other systems. In *S. cerevisiae*, inactivation of a temperature sensitive mutant of Ulp1, a SUMO protease, prevents the nuclear retention of unspliced mRNAs [50]. Moreover, several yeast TREX components are known to be sumoylated [51,52,53]. Indeed, sumoylation enzymes genetically interact with many genes involved in mRNA processing and nuclear export in yeast [54]. In plants, the connection between sumoylation and mRNA export is even more apparent. Mutations in either the SUMO E3 conjugating enzyme Siz1 or the SUMO protease ESD4 causes poly(A) accumulation in the nuclei of *A. thaliana* seedling cells [55]. Moreover, plants with mutations in certain mRNA export factors also tend to accumulate an excess of poly-sumoylated conjugates [40]. Although sumoylation has yet to be linked to mRNA nuclear export in metazoans, many mRNA binding proteins are known to be sumoylated and thus may be regulated by this post-translational modification [56,57].

Although our data indicates that sumoylation is required for the long-term cytoplasmic accumulation of certain mRNAs, it does not seem to play a direct role in mRNA nuclear export. Instead, our results are consistent with the idea that the production of certain transcripts in sumoylation-deficient cells leads to a reorganization of the nucleo-cytoplasmic space. Since all of the encoded polypeptide of *MHC-ftz-*Δ*i* is secreted [21], it is likely that the mRNA itself, rather than its protein product, is the culprit. While the reasons for this are unclear at the moment, it is possible that in the absence of sumoylation, certain mRNP components are inefficiently recycled back into the nucleus where they are used for the subsequent assembly of new mRNP complexes. This model is in line with various reports linking sumoylation to nuclear-cytoplasmic transport events [58]. Interestingly, this effect is only seen with certain mRNAs. Since this outcome is eliminated when introns are inserted into our overexpressed reporter, it suggests that the defective cycling of mRNP components is ameliorated when the spliceosome can access the mRNPs. Interestingly, during spermatogenesis SC35-foci start to disappear from the nucleus and appear in the cytoplasm. This reorganization seems to be linked to a decrease in RanBP2 levels [59]. Although depletion of RanBP2 in our system does not lead to either the appearance of SC35-foci in the cytoplasm [49] or the nuclear retention of any mRNAs [21], it is nevertheless interesting that RanBP2 is a known E3 SUMO ligase, thus providing another link between sumoylation and nuclear speckle disruption.

We also present data consistent with the idea that GANP is required for the export of most mRNA. This finding agrees with previously published reports [12,13,14]. In recent work it has been suggested that GANP may primarily affect certain mRNAs [14]; however, since the authors of that study found an inverse correlation between dependence on GANP and mRNA stability, it is entirely possible that the distribution of long-lived mRNAs are naturally less susceptible to changes in nuclear export kinetics.

Our study is a good example of why performing nuclear microinjection studies is critical in order to assess mRNA nuclear export. The long-term overexpression of a reporter mRNA may eventually incapacitate the entire mRNA export pathway in certain circumstances. Although these studies can be useful in identifying cellular states where the mRNA export pathway can be overwhelmed, they also can be misinterpreted.

Finally it is curious that we only recuperated two hits in our screen of mRNA export factors, since similar screens performed in insect tissue culture cells revealed as many as 72 different genes required for bulk mRNA export [60]. One problem with performing shRNA screens in mammalian cells is the effectiveness of the depletion. In the course of previous studies (for example [8,21,34]) and in this work (see Table S1, and [49]), only 60%–70% of lentiviral delivered shRNAs deplete their target when assayed by western blot. Thus it is likely that we may have missed additional targets due to ineffective RNAi. It is possible that many of the known nuclear export factors are only required for export of certain mRNAs or that their requirement vary between different cell types. However, it may also be the case that mRNA nuclear export pathways are extremely robust and employ many components that can compensate for each other’s losses. It has been well established that depletion in certain mRNA export factors leads to the upregulation of other export components [18]. It has also been previously found that several components of the TREX complex must be depleted before mRNA export defects can be observed in metazoans [17,22,24]. Obviously additional research will be needed to gain a clearer understanding of these mRNA export pathway components.

## 5. Conclusions

The overexpression of certain intronless mRNAs in cells that are depleted of the SUMO E2-ligase Ubc9 causes the redistribution of nuclear speckle markers to the cytoplasm and an inhibition of mRNA nuclear export.

## Supplementary Files

Supplementary File 1

## Abbreviations

ALPP: placental alkaline phosphatase
ALREX: alternative mRNA export
CALR: calreticulin
FISH: fluorescent *in situ* hybridization
GANP: germinal center-associated nuclear protein
H1B-GFP: histone 1B-GFP
INS: insulin
INSL3: insulin-like 3
mRNP: mRNA ribonucleoprotein
SSCR: signal sequence coding region
shRNA: short hairpin RNA
TREX: transcription export
